# The Cultural Tapestry of Tobacco Use in India: A Narrative Review

**DOI:** 10.7759/cureus.86884

**Published:** 2025-06-27

**Authors:** Saudamini More, Sanpreet S Sachdev, Numa Bareen, Laresh N Mistry, Amit Patil, Tejal Patil

**Affiliations:** 1 Department of Public Health Dentistry, Bharati Vidyapeeth (Deemed to be University) Dental College and Hospital, Navi Mumbai, IND; 2 Department of Oral Pathology and Microbiology, Bharati Vidyapeeth (Deemed to be University) Dental College and Hospital, Navi Mumbai, IND; 3 Department of Pediatric and Preventive Dentistry, Bharati Vidyapeeth (Deemed to be University) Dental College and Hospital, Navi Mumbai, IND; 4 Department of Conservative Dentistry and Endodontics, Bharati Vidyapeeth (Deemed to be University) Dental College and Hospital, Navi Mumbai, IND; 5 Department of Oral and Maxillofacial Surgery, Bharati Vidyapeeth (Deemed to be University) Dental College and Hospital, Navi Mumbai, IND

**Keywords:** india, tobacco

## Abstract

In India, both smoking and nonsmoking forms of tobacco are deeply ingrained in cultural and regional customs. Being the largest producer and consumer of tobacco, India witnesses a wide variety of cigarettes, bidis, hookah, chilam, ghutka, pan masala, khaini, and betel quid with tobacco. The consumption of these products is based on sociocultural notions with variations across education, gender, and geography. Historically, smoking is linked with masculinity and authority, especially in rural areas. At the same time, female tobacco use is prevalent in neighboring countries like Bangladesh despite being stigmatized in Indian society. There, women cite oral health benefits and enhanced concentration as justification for consumption.

The multifactorial behavior of tobacco consumption is influenced by social and cultural norms and practices. Hookah, cigars, and shisha often symbolize status in social gatherings. In the face of growing knowledge and awareness of its health hazards, strong association with oral precancerous lesions such as oral submucous fibrosis, erythroplakia, and leukoplakia, tobacco remains dangerously addictive and tough to quit. Significant geographic clustering was observed in different states and union territories of India, showing regional influences.

Public health initiatives in India should be culturally tailored and area-specific, owing to the strong tobacco habits and cultural underpinnings. Targeted policy modifications that are cognizant of local customs and habits may boost the impact and efficacy of tobacco control programs and substantially lower the nationwide tobacco-related health burdens.

## Introduction and background

Tobacco, a plant with deep roots in human history, has left a complex imprint on cultures around the world. In a vast country like India, we find that tobacco is consumed in varied smoking and nonsmoking forms. India is the largest producer and consumer of tobacco. Smoking tobacco includes hookah, cigarettes, bidis, charas, and so on, while on the other hand, nonsmoking tobacco products include pan, pan masala, gutkha, gul manjan, surti, supari, and khaini [1]. According to the Global Adult Tobacco Survey 2016-2017, approximately 28.6% of Indian adults consume tobacco in some form, with 42.4% of men and 14.2% of women being current users. Smokeless tobacco (SLT) use stands at 21.38%, while smoking tobacco use is at 10.38% [2].

In India, tobacco has been intricately associated with tradition, social behavior, economic practices, and religious customs. While modern health concerns now dominate conversations about tobacco, its cultural significance in India spans centuries and continues to influence attitudes and behaviors today.

Tobacco is not indigenous to India; it was introduced by the Portuguese in the 16th century. However, its assimilation into Indian culture was swift. Within decades, tobacco found its way into royal courts, local rituals, and rural communities [3]. Early historical records reveal that smoking and chewing tobacco were prevalent among the nobility and commoners alike. Hookah smoking, for instance, became fashionable among Mughal emperors and aristocrats, often symbolizing refinement and leisure. Over time, this aristocratic practice trickled down into various layers of society and adapted to local customs [4]. By the 18th century, India had developed diverse forms of tobacco use such as smoking (bidi, hookah, and cigarette), chewing (khaini and gutkha), and snuffing (naswar). Each method carried its own cultural and social significance [5].

Although tobacco does not occupy a sacred status in mainstream Hinduism or other major Indian religions, it has been used in various ritualistic contexts, some of which are formal while others are folk-based. In rural India, especially in tribal and agrarian communities, tobacco has occasionally been used in folk rituals. In some regions, offerings of tobacco are made to appease local deities or spirits. Among certain tribal groups, tobacco is believed to have protective properties and is used in charms or as a ward against evil. Tobacco smoke is sometimes used in cleansing rituals to remove negative energy from a household or space [6,7]. The integration of tobacco into the practices of these spiritual figures contributes to its cultural legitimacy and normalization, especially in rural areas.

The present narrative review aims to collectively examine the multifaceted cultural aspects of tobacco in India, exploring its historical context, ritualistic and religious uses, regional variations, social symbolism, gender dynamics, and the tensions between tradition and modernity.

## Review

Methodology

A literature review was conducted using the keywords "Tobacco", "gutkha", "khaini", "cigarette", "bidi", "India", "North India", "West India", "South India", and "East India", in various combinations with Boolean operators "AND" and "OR". Searches were performed across multiple scholarly databases including PubMed, ScienceDirect, EBSCOHost, Web of Science, and Google Scholar to identify relevant peer-reviewed articles. Only studies published in the English language were considered, and no restrictions were placed on the year of publication. The search primarily focused on studies addressing the sociocultural, geographical, gender-based, and economic determinants of tobacco use in India. Cross-sectional studies, observational surveys, qualitative reports, and review articles examining regional habits and influencing factors were included. In contrast, articles limited to pharmacological effects, clinical trials, or biochemical mechanisms were excluded.

**Figure 1:**
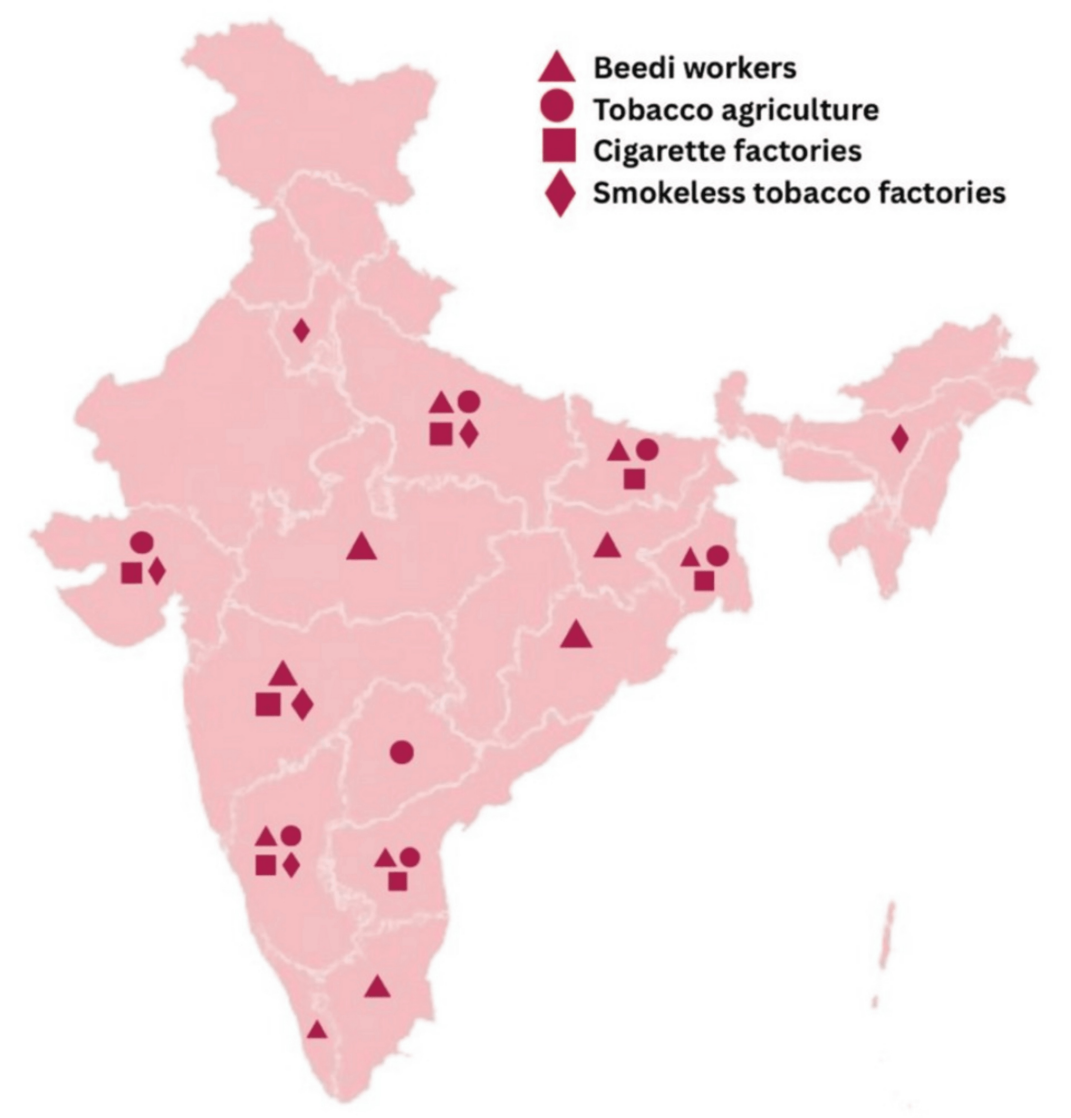
Preference of tobacco habits across different states of India Image credit: This is an original image created by the author Numa Bareen

The initial pool of literature was screened for relevance, and the final selection was based on thematic significance and regional representativeness. Among the studies reviewed, 10 publications were identified as especially illustrative of regional and sociocultural diversity, and these were compiled in a tabular form to demonstrate the breadth of findings across India’s geographical zones. The data were independently analyzed by the authors SM, SSS, and NB and were then thematically organized based on cultural relevance, regional usage patterns, gender-specific insights, and socioeconomic dimensions. A narrative synthesis was then performed, integrating these findings into structured sections corresponding to the country’s major regions. India’s vast diversity is reflected in the regional preferences (Figure 1) and practices associated with tobacco.

Regional-cultural variations of tobacco forms

Northern India

Hookah smoking is especially popular in Haryana, Punjab, and parts of Uttar Pradesh. Often a communal activity, it is associated with hospitality and village gatherings. In rural settings, offering a puff from a hookah can be a sign of trust and friendship [8].

A study focusing on Northern India reported that among 1,607 tobacco users, 31.8% used chewing tobacco, 16.55% smoked, and a significant 52.15% engaged in both smoking and chewing. The most commonly used products included tobacco-betel, gutkha, cigarettes, bidis, and khaini [9]. The use of tobacco in North India is influenced by cultural practices and social norms. Products like gutkha and khaini are often integrated into daily routines, making cessation efforts challenging [10].

Eastern India

Khaini (a mixture of tobacco and slaked lime) is commonly used in Bihar, Jharkhand, and parts of Odisha. The practice is often gendered, with both men and women using khaini discreetly. Smoked tobacco includes bidis (hand-rolled cigarettes), cigarettes, and hookahs.​ SLT products, such as khaini (tobacco mixed with slaked lime), gutkha, and pan masala, are widely used [11]. A meta-analysis covering data from 2010 to 2022 revealed that the East zone of India has the highest prevalence of tobacco use at 55.4%, followed closely by the Northeast at 51.8%. SLT use is particularly prevalent among women in the Northeast, with a study noting a 23% usage rate among women in these states [12,13].

Western India

Gutkha and pan with tobacco are widely consumed in Maharashtra and Gujarat. These preparations are deeply embedded in social customs, used in celebrations, weddings, and daily life [14]. Products like gutkha, khaini, and mawa are widely consumed. Mawa, a mixture of areca nut, tobacco, and lime, is especially popular in these western states [15].

South India

Tobacco consumption in South India exhibits notable regional and demographic variations, with patterns influenced by factors such as gender, socioeconomic status, and urban-rural residence [16]. In South India, the prevalence of any tobacco use was 20.0%, with smoking at 9.9% and SLT use at 4.8%. Tobacco use was more prevalent among men, individuals with lower education levels, and those from rural areas [17]. Tobacco is frequently chewed with betel leaf (pan) in states like Tamil Nadu, Andhra Pradesh, and Karnataka. Snuffing tobacco is also prevalent among older populations [6,10, 11].

A community-based study in rural Tamil Nadu focusing on women aged 18 and above found that 15.2% of participants consumed tobacco, exclusively in smokeless forms [18]. A significant majority (87.5%) expressed unwillingness to quit, citing tobacco as an integral part of their daily routine.​ A survey conducted in and around Chennai city reported an overall tobacco use prevalence of 21%, with higher rates in rural areas compared to semiurban and urban locales [19].

Region-Specific Plan of Action

Regional assessments of tobacco usage are based on prevalence data, gender disparities, product types, ritualistic uses, and socioeconomic affiliations. These dimensions serve as critical indicators to guide region-specific intervention strategies. To tackle these, it is essential to devise certain plans of action specific to those regions (Table 1).

**Table 1:** Targeted actions for each region SLT: smokeless tobacco

Region	Key tobacco form	Cultural anchor	Suggested action plan
North India	Hookah, gutkha	Hospitality, masculinity	Engage local panchayats, offer cessation in social centers
East India	Khaini, bidi	Daily routine, gender-equal	Female-targeted education and discreet cessation tools
South India	Pan with tobacco	Socioeconomic burden	Rural outreach + alternate livelihoods
Northeast	High female SLT use	Social tradition	Gender-sensitive campaigns + tribal community involvement

Various aspects of social symbolism

Tobacco use in India carries various symbolic meanings, depending on context and demographic group.

Authority and Hospitality

In many rural communities, smoking or chewing tobacco is associated with masculinity, strength, and authority. Elders and community leaders often use tobacco publicly as a display of status [20]. Boys see their grandfathers or fathers smoking, so they think it is part of being a man [4]. Smoking is seen as part of being a man and a sign of his male authority. Tobacco, especially in the form of hookah or pan, is a medium of hospitality. Offering tobacco to guests signifies warmth and social acceptance [21].

Caste and Class Dynamics

Historically, access to certain forms of tobacco was stratified. Hookah smoking was reserved for the upper castes and landowners [22]. Bidis and SLT were more common among lower castes and working classes [23]. These associations have eroded over time, but the residual perceptions still linger in certain areas.

Gender and Tobacco Use

Tobacco use among Indian women has long been stigmatized in urban areas, but this dynamic changes significantly in rural and tribal communities. In many rural parts of India, especially in the Northeast, women openly chew tobacco or use snuff, and it is not seen as taboo. For example, in Assam and Nagaland, tobacco is used communally by women during social gatherings. Among tribal women in Madhya Pradesh and Chhattisgarh, SLT is considered part of everyday life [10,24]. In contrast, urban women who smoke or chew tobacco often face social judgment. However, this stigma is slowly changing with increased gender autonomy, changing norms, and media influence.

Sociocultural Aspects and Influence of Media

National and regional cinema have played a complex role in both glamorizing and problematizing tobacco use. For decades, cigarette smoking by male leads in Indian cinema symbolized rebellion, power, and seduction. Iconic stars, including various male superstars, have smoked on screen, normalizing the practice [25]. Table 2 comprehensively describes the studies conducted regarding the sociocultural aspects of tobacco habits in India.

**Table 2:** Studies highlighting the sociocultural aspect of tobacco habit in India SLT: smokeless tobacco; UTs: union territories

Sr. no.	Year	Region of India	Form of tobacco	Cultural importance and habits	Conclusion
1	Farooqui et al. [1]	Urban and rural India	Cigarettes, bidis, and SLT	Social and cultural motivations; peer influence	High prevalence linked to sociocultural factors; awareness programs needed
2	Pahari et al. [11]	Nationwide	Cigarettes, bidis, and SLT	Regional disparities; cultural factors influence use	East and Northeast have highest prevalence; regional focus needed
3	Singh et al. [26]	Nationwide (30 states and two UTs)	Cigarettes, bidis, smokeless, and dual use	Local clustering; influenced by area-level factors	Tobacco use is highly clustered geographically; local policies needed
4	Murmu et al. [27]	Eastern India; tribal populations	SLT and smoking	Cultural norms among tribal groups; alcohol co-use	High SLT use among tribes; tailored interventions required
5	Verma et al. [28]	Northern and Northeastern India	Hookah, bidis, and SLT	Hookah as a social ritual; early initiation in rural areas	Cultural practices influence early initiation; need for targeted interventions
6	Bantwal et al. [29]	Northeast and Central India	Cigarettes, bidis, and SLT	Poly-tobacco use is prevalent among disadvantaged groups	High poly-tobacco use; focus on vulnerable populations needed
7	Aluckal et al. [30]	Kerala (Kuttampuzha tribal area)	Bidis and SLT	Deep-rooted in tribal culture; low awareness	Cultural norms hinder cessation; need for culturally sensitive programs
8	Goyal et al. [31]	Nationwide	SLT and bidis	Cultural acceptance in certain regions; gender norms	SLT use is prevalent among women; gender-specific interventions required
9	Shah et al. [32]	Nationwide	Cigarettes, bidis, and SLT	Socioeconomic disparities; cultural practices	Tobacco use varies with socioeconomic status; targeted policies needed
10	Bhatt et al. [33]	Punjab	Cigarettes and SLT	Sikhism prohibits tobacco; underreporting due to stigma	Cultural beliefs affect reporting; culturally tailored interventions necessary

In recent years, the narrative has shifted. Public health campaigns have pushed for tobacco disclaimers before films. Movies now portray the consequences of tobacco use, aligning with national health messages [34,35]. Still, the deep cultural embedding of tobacco makes its portrayal a sensitive issue in media regulation [36].

Health, tradition, and modern tensions

India faces a paradox: while tobacco is culturally embedded, it is also a public health crisis. India has the second-largest population of tobacco users in the world and a high burden of tobacco-related illnesses [37,38]. In areas where tobacco is linked to identity, livelihood, and tradition, public health campaigns often face resistance. For example, banning gutkha in some states led to the rise of illicit markets rather than behavioral change. Campaigns are often perceived as urban, elite interventions lacking cultural sensitivity [39]. Recent health initiatives have started incorporating cultural insights by engaging local leaders and storytellers in rural tobacco control. Framing tobacco cessation is a way to protect family and community, rather than just individual health [40].

Tobacco is not just a substance of consumption; it is a livelihood for millions in India. Thus, it contributes to the economic and cultural independence of the country. India is a significant producer of tobacco, particularly in states such as Andhra Pradesh, Karnataka, and Gujarat. For many small farmers, tobacco is a cash crop [10,41]. Bidi rolling and gutkha production employ millions, particularly women, in informal settings. In such contexts, tobacco is deeply tied to survival and family economics, complicating public health efforts [42].

Translational implications

The insights presented in this narrative review underscore the necessity for culturally grounded approaches to tobacco control in India. Given the profound regional, gendered, and sociocultural embedding of tobacco use, a one-size-fits-all strategy is unlikely to be effective. Translating these findings into public health action requires tailoring interventions that resonate with local identities, customs, and lived experiences.

Public health campaigns must move beyond generic messaging to incorporate cultural symbolism, oral traditions, and community hierarchies, such as engaging village elders, local panchayats, and tribal leaders as champions of antitobacco advocacy. Integration of tobacco cessation into existing rural healthcare systems, including training Accredited Social Health Activists and Anganwadi workers in culturally sensitive counseling, is essential.

In urban and semiurban areas, the use of digital platforms and regionally produced media content can reshape norms around tobacco consumption. Furthermore, education programs in schools should include context-specific modules on the social and health impacts of tobacco, particularly in regions where early initiation is prevalent.

Economic alternatives and rehabilitation programs must be developed for communities reliant on bidi rolling or gutkha production, especially for women in informal labor sectors. Finally, policy initiatives should embed cultural diagnostics such as the prevalence of communal tobacco practices or caste-linked consumption patterns into the design of regulations, taxation, and surveillance systems. Only by embedding these culturally nuanced findings into tangible policy frameworks and community programs can India hope to mitigate the complex burden of tobacco use across its diverse populations.

Implementation strategies

To effectively address the culturally entrenched nature of tobacco use in India, multitiered and locally responsive implementation strategies are essential. First, region-specific interventions should be designed using local epidemiological data and cultural practices.

For example, traditional community gatherings can be incorporated in North India (where hookah is socially symbolic) into cessation outreach programs led by respected village elders. In tribal belts of Eastern and Central India, where SLT use is normalized among both genders, community health workers should be trained to integrate tobacco cessation advice into maternal and child health visits and immunization drives. In South India, where women engaged in bidi rolling form a vulnerable labor force, state-supported livelihood substitution schemes and microfinancing options can gradually shift economic dependence away from tobacco-based cottage industries.

School-based educational curricula, especially in rural and semiurban zones, should be redesigned to reflect local tobacco forms and include culturally relatable content, possibly in local dialects or folk formats. Mobile health (mHealth) platforms and social media can also be leveraged to promote behavioral change among youth, especially in urban slums where traditional norms are intersecting with modern media influences [43].

Furthermore, state and district-level tobacco control programs should allocate funding for culturally sensitive Information, Education, Communication materials, featuring regionally popular idioms, visuals, and testimonials. Monitoring and evaluation frameworks must incorporate qualitative assessments of community perceptions, adoption barriers, and changes in social acceptability, thereby ensuring that interventions remain adaptive and contextually relevant over time.

## Conclusions

The tobacco habit in India is embedded socioculturally. It carries centuries of tradition, symbolism, and social meaning. From rituals and caste identity to gender dynamics and economic sustenance, tobacco is woven into the Indian cultural landscape in intricate ways. Efforts to reduce tobacco use in India must therefore go beyond regulatory frameworks. Policy interventions should be directed to influence specific local sociocultural factors on adult tobacco use so as to be effective in India. Tobacco habits in India are specific to the region and form a large part of the tradition. Tailoring tobacco control policies for local areas in India may, therefore, provide substantial public health benefits. Balancing public health with cultural sensitivity remains a critical challenge and an opportunity for India as it moves toward a tobacco-free future.
